# The impact of potentially modifiable risk factors for stroke in a middle-income area of China: A case-control study

**DOI:** 10.3389/fpubh.2022.815579

**Published:** 2022-08-19

**Authors:** Yuhang Wu, Xiaoyun Chen, Songbo Hu, Huilie Zheng, Yiying Chen, Jie Liu, Yan Xu, Xiaona Chen, Liping Zhu, Wei Yan

**Affiliations:** ^1^Jiangxi Center for Disease Control and Prevention Institute of Chronic Non-communicable Diseases, Nanchang, China; ^2^Jiangxi Province Key Laboratory of Preventive Medicine, School of Public Health, Nanchang University, Nanchang, Jiangxi, China; ^3^Department of Epidemiology and Health Statistics, Xiangya School of Public Health, Central South University, Changsha, China

**Keywords:** stroke, risk factors, case-control study, population attributable risks, Chinese

## Abstract

**Aims:**

To reveal the impact of eleven risk factors on stroke and provide estimates of the prevention potential.

**Methods:**

We completed a multicenter case-control study in Jiangxi, China, a middle-income area. Neuroimaging examination was performed in all cases. Controls were stroke-free adults recruited from the community in the case concentration area. Conditional logistic regression and unconditional logistic regression were used for subgroup analysis of stroke type, and other groups (sex, age and urban-rural area), respectively. Odds ratios (ORs) and their population attributable risks (PARs) were calculated, with 95% confidence intervals.

**Results:**

A total of 43,615 participants (11,735 cases and 31,880 controls) were recruited from February to September 2018, of whom we enrolled 11,729 case-control pairs. Physical inactivity [PAR 69.5% (66.9–71.9%)] and hypertension [53.4% (49.8–56.8%)] were two major risk factors for stroke, followed by high salt intake [23.9% (20.5–27.3%)], dyslipidemia [20.5% (17.1–24.0%)], meat-based diet [17.5% (14.9–20.4%)], diabetes [7.7% (5.9–9.7%)], cardiac causes [5.3% (4.0–6.7%)], alcohol intake [4.7% (0.2–10.0%)], and high homocysteine [4.3% (1.4–7.4%)]. Nine of these factors were associated with ischemic stroke, and five were associated with intracerebral hemorrhage. Collectively, eleven risk factors accounted for 59.9% of the PAR for all stroke (ischemic stroke: 61.0%; intracerebral hemorrhage: 46.5%), and were consistent across sex (men: 65.5%; women: 62.3%), age (≤55: 65.2%; >55: 63.5%), and urban-rural areas (city: 62.2%; county: 65.7%).

**Conclusion:**

The 11 risk factors associated with stroke identified will provide an important reference for evidence-based planning for stroke prevention in middle-income areas. There is an urgent need to improve awareness, management and control of behavioral and metabolic risk factors, particularly to promote physical activity and reduce blood pressure.

## Introduction

Stroke is the leading cause of death and disability worldwide (1, 2). China accounts for nearly one-third of all deaths from stroke worldwide (3). China is one of the countries with the heaviest stroke burden in the world (4). Stroke became the leading cause of years of life lost (YLLs), and stroke was the leading cause of all-age disability-adjusted life-years (DALYs) in 2017 (5). In the past 30 years, the burden of stroke in China has been increasing, especially in rural areas (4).

Prevention may be the most effective way to reduce this burden of stroke, making it a global health priority for changing the status of stroke (6). It is necessary to identify and quantify the modifiable and common risk factors for stroke. Prior to this study, the Global Burden of Disease Study (GBD) reported regional differences in the burden of stroke by modifiable risk factors, including 188 countries around the world, but there is a lack of discussion specific to China (7). The INTERSTROKE study is an international, multicenter, case-control study, designed to establish the association of traditional and emerging risk factors with stroke (and primary stroke subtypes) in countries of high, middle, and low income, and the completion of two phased results shows that a case-control study is feasible for stroke (8, 9). In the recent report of phase 2, participants were recruited from 142 centers in 32 countries around the world, and it reported that ten potentially modifiable risk factors were collectively associated with more than 90% of the population attributable risks (PARs) of stroke in each major region of the world, and there were regional differences in stroke types and differences in leading risk factors by age and sex on a global scale (including China) (8). However, a major limitation of previous research is that the estimated combined PAR makes the assumption of independence of the risk factors untenable, as some risk factors may be intertwined (8). For example, hypertension may be related to obesity and diabetes, whereas people with hypertension, obesity, and diabetes are more prone to dyslipidemia, constituting metabolic syndrome, which is related to physical inactivity. Therefore, the combined PAR is likely to be severely overestimated. In addition, the INTERSTROKE results showed that some important risk factors, such as diabetes and cardiac causes, were not significantly associated with stroke, and physical inactivity rather than hypertension was the largest risk factor for stroke in China, which was inconsistent with results in other parts of the world (8).

As China is an extremely populous country that accounts for one-fifth of the world's population, there are still differences in health issues and access to health care providers and services among provinces, and the differences are gradually being widened. Evidence-based health decision-making at the provincial level is crucial (5, 10). To our knowledge, evidence to determine the relative importance of risk factors associated with stroke is still lacking, especially in low-income and middle-income areas with a high burden of stroke in China. Based on the INTERSTROKE study (8, 9), Jiangxi Province, a region in the southeastern part of mainland China with a per capita gross domestic product (GDP) level that was at the national average, was selected to quantify the impact of modifiable risk factors for stroke. This study aimed to identify and determine stroke risk factors that may uniquely contribute to the Chinese population by using combined PAR adjusted for the nonindependence of risk factors. We will further propose to add subgroup analyses, such as urban and rural areas, and to include homocysteine as a new risk factor based on the available relevant evidence (11, 12).

## Materials and methods

### Study design and participants

This study was supported by the National Stroke Center's screening and intervention project for individuals at high risk of stroke, which was conducted on the basis of research centers with stroke cases and communities (townships) with residents. The number of respondents obtained from Jiangxi Province was determined to be ~48,000, and the ratio of research center to community (township) was 1:2. The stroke cases were recruited from eight research centers (Xinyu People's Hospital, the Second Affiliated Hospital of Nanchang University, Pingxiang People's Hospital, Jiujiang No. 1 People's Hospital, Jingdezhen No. 1 People's Hospital, Ganzhou People's Hospital, Yichun People's Hospital and Jiangxi Provincial People's Hospital) selected by the National Stroke Center in Jiangxi Province, China, from February to September 2018. Neuroimaging examinations were completed in all cases. Stroke was defined using the World Health Organization clinical criteria for stroke (13). All the survey information was answered by the patients themselves or by the patients and their first-degree relatives together. We included both ischemic stroke and intracerebral hemorrhage in this study. The type of stroke cases was based on clinical assessment and CT or MRI, electrocardiogram, cerebral angiography, and carotid ultrasound in accordance with standard operating procedures. Moreover, case records with unclear diagnosis and classification of stroke type were excluded from this study. The controls were permanent residents without stroke who had lived in the investigation site for more than 6 months and were 40 years of age or older; they were all from the communities in the catchment areas or nearby areas of the hospitals where cases were recruited. Sixteen counties (cities, districts) were randomly selected using a multistage cluster sampling method, and the number of controls in each county (city, district) was required to be no <2,000. The stroke status for controls was comprehensively judged and ruled out by the neurologist during the interview and investigation by asking about the history of stroke, neurological symptoms and signs, and auxiliary examinations.

Although investigators who were trained and assessed used different questionnaires developed by the National Stroke Center for cases and controls to collect information, the information on the questionnaires for the risk factors involved in this study was consistent. Laboratory inspections were quickly tested by instruments, and all test results were automatically recorded. Information on variables included in this study was obtained during recruitment through inquiries, laboratory inspections, and hospital original records. The data were collated and cleaned by the National Health Commission of the People's Republic of China, the National Stroke Center, and the Chinese Center for Disease Control and Prevention. Each control was matched for sex and age with cases, and age matching was extended (±5 years) for a few participants younger than 45 years and older than 90 years.

### Definitions of risk factors

Cardiac disease was defined as a history of atrial fibrillation, cardiomyopathy, heart failure, ischemic heart disease, rheumatic heart disease, or valvular disease after diagnosis by doctors in secondary or higher hospitals or when the ECG showed abnormalities. Hypertension was defined as having a history of being diagnosed with hypertension by a secondary or higher hospital or blood pressure (mean of three measurements) of 140/90 mm Hg or higher. Blood pressure was measured at the time of admission. Diabetes was defined as a history of diabetes or a fasting blood glucose concentration >7.0 mmol/L at the first encounter. Smoking status was defined as cumulative smoking for more than 6 months in a lifetime (current smoking and former smoking). Alcohol intake was classified as never, low or moderate intake and high (more than three times a week and 100 ml each time) intake. Physically active individuals were defined as those involved in moderate or strenuous activity three times or more and 0.5 h or more per week, or those engaged in moderate or heavy physical labor (14). High salt intake and a meat-based diet were defined by self-reported daily diet preference for salty taste and preference for meat, respectively (14). For obesity, we assessed body mass index (BMI). Individuals with BMI ≥ 30 were defined as obese (15). Dyslipidemia was defined according to the Chinese guidelines for the prevention and treatment of dyslipidemia in adults as follows (16): triglyceride (TG) ≥ 2.26 mmol/L; total cholesterol (TC) ≥ 6.22 mmol/L; low-density lipoprotein cholesterol (LDL-C) ≥ 4.14 mmol/L; and high-density lipoprotein cholesterol (HDL-C) <1.04 mmol/L. According to the WHO standard, the average level of homocysteine for healthy adults is 5–15 μmol/L, with a homocysteine level > 15 μmol/L representing high homocysteine (17). The questionnaire items, with their answer options for some risk factors involved in the study, are described in Supplementary Table 1.

### Statistical analysis

We used the McNemar χ^2^ test to assess the bivariate correlation between risk factors and stroke status. The age, sex, and urban-rural differences between matched cases and controls were compared with paired *t*-tests. Controls were matched with the cases in a 1:1 ratio, and we used conditional logistic regression for the primary analysis of all strokes. A similar analysis was conducted and stratified by stroke type. We also used unconditional logistic regression to establish an association between risk factors and stroke by sex (men vs. women), age groups (≤55 vs. >55 years) and urban-rural areas (city vs. county). The participants were classified into urban populations and rural populations based on their domicile locations. All conditional regression analyses were adjusted according to age, sex and urban-rural areas. We also performed a sensitivity analysis using different definitions for obesity.

Estimates of the odds ratios (ORs) and 95% confidence interval (95% CI) in the final models were presented for every risk factor. We calculated the PAR for each risk factor using Levin's formula (18). The combined estimate of the PAR assumed independence of risk factors using this formula:


$$\begin{array}{l} PAR=1-\left[\left(1-PAR_{1}\right)\left(1-PAR_{2}\right)\left(1-PAR_{3}\right)\ldots \right] \end{array}$$

To explain the nonindependence of risk factors, weighting was included in the calculation of the overall PAR using the following formula:


$$\begin{array}{l} PAR_{adjusted}=1-[(1-w_{1}\times PAR_{1})(1-w_{2}\times PAR_{2})\;\\ \;(1-w_{3}\times PAR_{3})\ldots ] \end{array}$$

where the weight w was 1 minus the communality of each risk factor. The communality was calculated as the sum of the square of all factor loadings *via* principal component analysis of the inter-risk-factor correlation matrix (19). Statistical analyses and graphics were produced with the R statistical program (version 4.0.3).

## Results

A total of 43,615 participants were recruited from February to September 2018, comprising 11,735 cases of stroke and 31,880 controls. We identified 11,729 cases (9,880 with ischemic stroke and 1,849 intracerebral hemorrhage) and 11,729 controls according to case-control matching by sex and age, and six cases were excluded due to a failed match. There was no significant difference between the case group and the control group in the dimensions of sex, age and urban-rural areas after matching. The demographic and clinical characteristics of the cases are reported in Supplementary Table 2. A total of 6,801 (58.0%) participants in each group were men, and the mean age was 66.76 years (SD = 11.91) for cases vs. 66.65 years (11.49) for controls. A total of 5,568 (47.5%) participants in the case group lived in the city. Neuroimaging examinations were completed in all cases. Questionnaire surveys were completed by patients and their families. In total, 1,994 (17.0%) patients had cerebral angiography, and 5,290 (45.1%) had carotid ultrasound.

**Figure 1:**
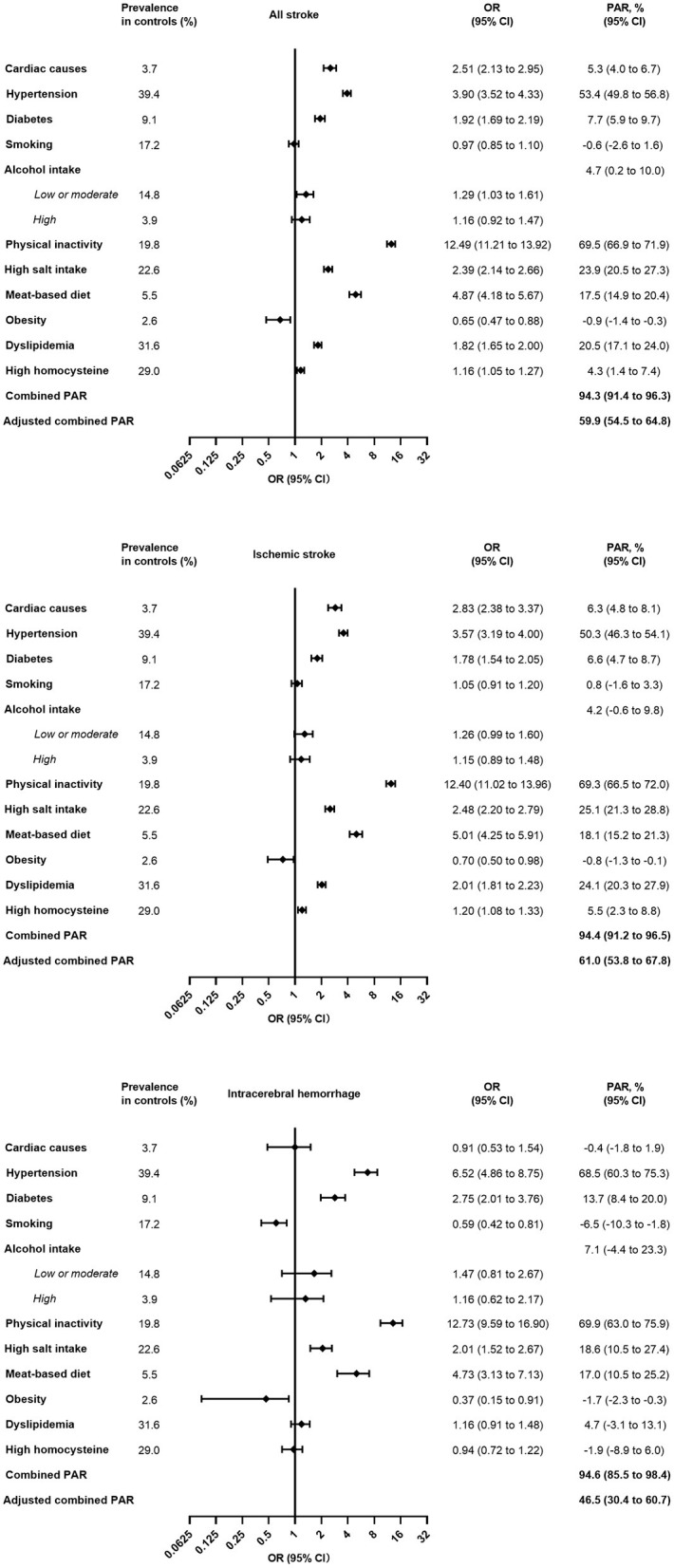
Multivariable analysis of prevalence of risk factors, OR, and PAR for eleven risk factors. OR, odds ratio; PAR, population attributable risk. For alcohol intake, PAR was calculated using low or moderate + high vs. never.

Eleven risk factors associated with stroke, ischemic stroke, and intracerebral hemorrhage are presented in Figure 1. Cardiac causes, hypertension, diabetes, physical inactivity, high salt intake, a meat-based diet, dyslipidemia and high homocysteine were associated with all strokes. The same risk factors were also associated with ischemic stroke. Hypertension, diabetes, physical inactivity, high salt intake and a meat-based diet were all independent predictors of intracerebral hemorrhage. Physical inactivity [PAR 69.5% (66.9–71.9%)] and hypertension [53.4% (49.8–56.8%)] were two major risk factors for stroke. Hypertension was associated with a larger OR in intracerebral hemorrhage than ischemic stroke. These 11 factors combined accounted for 94.3% (95% CI 91.4–96.3), 94.4% (95% CI 91.2–96.5) and 94.6% (95% CI 85.5–98.4) of the combined PAR associated with all stroke, ischemic stroke and intracerebral hemorrhage, respectively. The adjusted combined PAR was 59.9% (54.5–64.8) for all strokes, 61.0% (53.8–67.8) for ischemic stroke, and 46.5% (30.4–60.7) for intracerebral hemorrhage.

The combined PARs and adjusted combined PARs were 96.2% (95% CI 94.2–97.6) and 65.5% (95% CI 60.7–69.8) for men and 95.1% (95% CI 93.1–96.6) and 62.3% (95% CI 58.2–66.4) for women (Table 1). The PAR of alcohol intake of men was higher than that in women because of the higher prevalence. Among women, a meat-based diet had a stronger association with stroke.

**Table 1:** Risk factors for all stroke in men and women.

	**Control**		**All stroke cases**			
	**Men (*N* = 12,894)**	**Women (*N* = 18,986)**	**Men (*****N*** = **6,807)**		**Women (*****N*** = **4,928)**	
			**OR (95% CI)**	**PAR (95% CI)**	**OR (95% CI)**	**PAR (95% CI)**
Cardiac causes	459/12,894 (3.6%)	735/18,986 (3.9%)	2.67 (2.28–3.13)	5.7% (4.4–7.1)	2.52 (2.18–2.92)	5.6% (4.4–7.0)
Hypertension	5,130/12,894 (39.8%)	7,429/18,986 (39.1%)	5.13 (4.68–5.61)	62.1% (59.4–64.7)	4.95 (4.50–5.44)	60.7% (57.8–63.4)
Diabetes	1,171/12,894 (9.1%)	1,721/18,986 (9.1%)	1.99 (1.77–2.23)	8.3% (6.6–10.1)	2.03 (1.81–2.27)	8.6% (6.9–10.4)
Smoking	5,162/12,894 (40.0%)	324/18,986 (1.7%)	0.92 (0.84–1.00)	−3.4% (−6.8 to 0.1)	1.23 (0.94–1.60)	0.4% (−0.1 to 1.0)
Alcohol intake	–	–	–	12.7% (6.6–19.3)	–	−2.1% (−4.7 to −1.8)
Low or moderate	3,248/12,894 (25.2%)	1,462/18,986 (7.7%)	1.53 (1.30–1.79)	–	0.72 (0.44–1.18)	–
High	1,155/12,894 (9.0%)	98/18,986 (0.5%)	1.11 (0.94–1.33)	–	1.13 (0.68–1.87)	–
Physical inactivity	2,412/12,894 (18.7%)	3,898/18,986 (20.5%)	14.28 (13.12–15.55)	71.3% (69.4–73.1)	12.37 (11.31–13.53)	70.0% (67.9–72.0)
High salt intake	3,218/12,894 (25.0%)	3,982/18,986 (21.0%)	2.34 (2.13–2.57)	25.1% (22.0–28.1)	2.33 (2.11–2.56)	21.8% (18.9–24.6)
Meat-based diet	1,081/12,894 (8.4%)	677/18,986 (3.6%)	3.64 (3.24–4.10)	18.2% (15.8–20.6)	7.57 (6.58–8.70)	19.1% (16.7–21.7)
Obesity	294/12,894 (2.3%)	550/18,986 (2.9%)	0.51 (0.38–0.67)	−1.2% (−1.4 to −0.8)	0.45 (0.34–0.59)	−1.6% (−2.0 to −1.2)
Dyslipidemia	4,414/12,894 (34.2%)	5,659/18,986 (29.8%)	1.78 (1.64–1.94)	21.1% (18.0–24.3)	1.80 (1.65–1.96)	19.2% (16.2–22.3)
High homocysteine	4,904/12,894 (39.8%)	4,331/18,986 (22.8%)	1.23 (1.13–1.33)	8.3% (4.8–11.7)	1.39 (1.27–1.52)	8.1% (5.7–10.7)
Combined PAR	–	–	–	96.2% (94.2–97.6)	–	95.1% (93.1–96.6)
Adjusted combined PAR	–	–	–	65.5% (60.7–69.8)	–	62.3% (58.2–66.4)

OR, odds ratio; PAR, population attributable risk. For alcohol intake, PAR was calculated using low or moderate + high vs. never.

The combined PARs and adjusted combined PARs for 11 risk factors were 95.2% (95% CI 92.1–97.2) and 65.2% (95% CI 59.8–70.3) for individuals aged 55 years or younger and 95.7% (95% CI 94.1–96.9) and 63.5% (95% CI 59.8–67.0) for those older than 55 years (Table 2). Hypertension, diabetes and high homocysteine were associated with a larger OR in individuals aged 55 years or younger than in individuals aged 55 and over, whereas physical inactivity had a stronger association with stroke in those older than 55.

**Table 2:** Risk factors for all stroke by age group.

	**Control**		**All stroke cases**			
	**≤55 (*N* = 11,856)**	**>55 (*N* = 20,024)**	≤**55 (*****N*** = **2,232)**		>**55 (*****N*** = **9,503)**	
			**OR (95% CI)**	**PAR (95% CI)**	**OR (95% CI)**	**PAR (95% CI)**
Cardiac causes	179/11,856 (1.5%)	1,015/20,024 (5.1%)	1.79 (1.30–2.48)	1.2% (0.4–2.2)	2.41 (2.15–2.70)	6.7% (5.5–8.0)
Hypertension	3,033/11,856 (25.6%)	9,526/20,024 (47.6%)	6.26 (5.52–7.09)	57.4% (53.6–60.9)	4.01 (3.71–4.33)	58.9% (56.3–61.3)
Diabetes	693/11,856 (5.8%)	2,199/20,024 (11.0%)	2.59 (2.17–3.08)	8.4% (6.4–10.8)	1.83 (1.67–2.00)	8.4% (6.9–9.9)
Smoking	1,882/11,856 (15.9%)	3,604/20,024 (18.0%)	1.23 (1.05–1.44)	3.5% (0.7–6.6)	1.21 (1.11–1.32)	3.6% (1.9–5.5)
Alcohol intake	–	–	–	7.1% (0.8–14.7)	–	2.8% (−0.7 to 6.7)
Low or moderate	1,722/11,856 (14.5%)	2,988/20,024 (14.9%)	1.44 (1.06–1.94)	–	1.12 (0.95–1.33)	–
High	404/11,856 (3.4%)	849/20,024 (4.2%)	1.33 (0.97–1.81)	–	1.23 (1.03–1.48)	–
Physical inactivity	2,392/11,856 (20.2%)	3,918/20,024 (19.6%)	10.87 (9.59–12.32)	66.6% (63.4–69.6)	14.17 (13.21–15.20)	72.1% (70.5–73.6)
High salt intake	3,087/11,856 (26.0%)	4,113/20,024 (20.5%)	2.18 (1.92–2.49)	23.5% (19.3–27.9)	2.41 (2.23–2.60)	22.4% (20.1–24.7)
Meat-based diet	711/11,856 (6.0%)	1,047/20,024 (5.2%)	4.95 (4.20–5.83)	19.1% (16.1–22.5)	5.31 (4.76–5.91)	18.3% (16.4–20.3)
Obesity	328/11,856 (2.8%)	516/20,024 (2.6%)	0.59 (0.42–0.83)	−1.2% (−1.6 to −0.5)	0.45 (0.36–0.58)	−1.4% (−1.7 to −1.1)
Dyslipidemia	3,762/11,856 (31.7%)	6,311/20,024 (31.5%)	1.99 (1.76–2.24)	23.8% (19.4–28.2)	1.85 (1.73–1.99)	21.1% (18.6–23.7)
High homocysteine	2,460/11,856 (20.7%)	6,775/20,024 (33.8%)	1.70 (1.48–1.94)	12.6% (9.1–16.3)	1.22 (1.14–1.31)	7.0% (4.5–9.5)
Combined PAR	–	–	–	95.2% (92.1–97.2)	–	95.7% (94.1–96.9)
Adjusted combined PAR	–	–	–	65.2% (59.8–70.3)	–	63.5% (59.8–67.0)

OR, odds ratio; PAR, population attributable risk. For alcohol intake, PAR was calculated using low or moderate + high vs. never.

The combined PARs and adjusted combined PARs were 96.0% (95% CI 94.1–97.3) and 62.6% (95% CI 57.9–66.3) for those who lived in urban areas and 95.7% (95% CI 93.9–97.1) and 65.8% (95% CI 61.7–69.5) for those who lived in rural areas, respectively (Table 3). Diabetes and high salt intake were more associated with stroke in people living in rural areas, whereas smoking, physical inactivity, and a meat-based diet were associated with stroke in those who lived in urban areas. Hypertension was associated with a larger OR in rural areas than in urban areas. In the subgroup analysis, we also explored some risk factors for ischemic stroke and intracerebral hemorrhage separately, with preservation of these similar results (Supplementary Tables 3–5).

**Table 3:** Risk factors for all stroke in urban and rural areas.

	**Control**		**All stroke cases**			
	**City (*N* = 15,642)**	**County (*N* = 16,238)**	**City (*****N*** = **5,572)**		**County (*****N*** = **6,163)**	
			**OR (95% CI)**	**PAR (95% CI)**	**OR (95% CI)**	**PAR (95% CI)**
Cardiac causes	776/15,642 (5.0%)	418/16,238 (2.6%)	2.26 (1.96–2.61)	5.9% (4.6–7.4)	3.00 (2.54–3.55)	4.9% (3.8–6.2)
Hypertension	6,565/15,642 (42.0%)	5,994/16,238 (36.9%)	4.44 (4.02–4.89)	59.1% (55.9–62.0)	5.53 (5.07–6.03)	62.6% (60.0–65.0)
Diabetes	1,744/15,642 (11.1%)	1,148/16,238 (7.1%)	1.55 (1.38–1.74)	5.8% (4.1–7.6)	2.65 (2.36–2.97)	10.5% (8.8–12.3)
Smoking	1,874/15,642 (12.0%)	3,612/16,238 (22.0%)	1.50 (1.38–1.69)	5.6% (3.8–7.6)	1.05 (0.95–1.16)	1.0% (−1.2 to 3.4)
Alcohol intake	–	–	–	0.0% (−3.2 to 3.8)	–	7.9% (2.8–13.7)
Low or moderate	1,839/15,642 (11.8%)	2,871/16,238 (17.7%)	0.97 (0.76–1.24)	...	1.38 (1.14–1.67)	...
High	350/15,642 (2.2%)	903/16,238 (5.6%)	1.15 (0.89–1.49)	...	1.29 (1.06–1.58)	...
Physical inactivity	2,604/15,642 (16.6%)	3,706/16,238 (22.8%)	17.45 (15.93–19.11)	73.2% (71.2–75.0)	10.14 (9.34–11.02)	67.6% (65.5–69.5)
High salt intake	2,354/15,642 (15.0%)	4,846/16,238 (29.8%)	2.27 (2.05–2.53)	16.0% (13.6–18.6)	2.28 (2.09–2.48)	27.6% (24.5–30.6)
Meat-based diet	1,081/15,642 (8.4%)	677/16,238 (3.6%)	6.02 (5.20–6.96)	29.6% (26.1–33.4)	4.71 (4.20–5.27)	11.8% (10.3–13.3)
Obesity	520/15,642 (3.3%)	324/16,238 (2.0%)	0.35 (0.27–0.47)	−2.2% (−2.5 to −1.8)	0.62 (0.47–0.82)	−0.8% (−1.1 to −0.4)
Dyslipidemia	5,506/15,642 (35.2%)	4,567/16,238 (28.1%)	1.65 (1.51–1.80)	18.6% (15.2–22.1)	1.99 (1.83–2.16)	21.7% (18.9–24.5)
High homocysteine	4,325/15,642 (27.6%)	4,910/16,238 (30.2%)	1.46 (1.33–1.60)	11.2% (8.3–14.1)	1.36 (1.25–1.48)	9.9% (7.1–12.7)
Combined PAR	–	–	–	96.0% (94.1–97.3)	–	95.7% (93.9–97.1)
Adjusted combined PAR	–	–	–	62.6% (57.9–66.3)	–	65.8% (61.7–69.5)

OR, odds ratio; PAR, population attributable risk. For alcohol intake, PAR was calculated using low or moderate + high vs. never.

## Discussion

In this study, we used an appropriate method to determine that 11 potentially modifiable and common risk factors were associated with ~60% of the adjusted PAR for stroke. These results were all lower than the unadjusted PAR reported by the GBD study and the INTERSTROKE study (over 90%) (7, 8), suggesting that there was a nonnegligible overlapping effect among the 11 risk factors. We further observed that the overall contribution of these 11 risk factors to stroke risk was the same between men and women, young and old people, and urban and rural areas, whereas the impact of individual independent factors on a specific population was not consistent.

The reported effects (size and direction) of some independent risk factors were different. Cardiac causes, dyslipidemia and high homocysteine were independent risk factors for ischemic stroke rather than intracerebral hemorrhage, which suggested that ischemic stroke can be prevented from a broader perspective. Inconsistent with many reports (7, 9, 15) in other regions of the world, physical inactivity rather than hypertension was identified as the most important risk factor in Jiangxi, China. This was the same as the results reported for China in the second phase of the INTERSTROKE study (8), which indicated that the priority of risk factors in different regions may be different. The PAR estimated from our study (69.5%) was higher than that estimated from the INTERSTROKE study (59.9%). It may be that the standards for meeting the completion of exercise were too strict in our design, which led to the excessive effect of the lack of physical inactivity and the occurrence of stroke. The greatest importance of physical inactivity to the occurrence of stroke (ischemic stroke and intracerebral hemorrhage) was retained after adjusting for age, sex and other factors. A more potent risk factor for urban areas than for rural areas is especially important in individuals aged 55 and over. Most people living in rural areas were engaged in agricultural work, and they may use the nature of work to replace daily physical activities to a certain extent, even if urban communities had better sports infrastructure (20). Inactivity rises with age (21, 22), and with the decline in physical functions, the elderly found it difficult to obtain adequate physical activity. Hypertension has also been identified as a very important risk factor for stroke. The estimated PAR for hypertension in our research (53.4%) was between the GBD study (64.1%) (7) and the INTERSTROKE study (47.9%) (8). We used the average of three blood pressure measurements to strive to keep measurement bias to a minimum compared with INTERSTROKE. In addition, the high cutoff value selected by the GBD study as the systolic blood pressure may overestimate the burden of blood pressure on stroke. Nonetheless, all results indicated that blood pressure control was an important goal for stroke prevention. The estimated PAR of ischemic stroke in hypertension (50.3%) was lower than that for intracerebral hemorrhage (68.5%) in our research due to the stronger association of blood pressure with intracerebral hemorrhage than ischemic stroke. Preventive measures such as raising awareness and managing blood pressure may be more effective in reducing the burden of intracerebral hemorrhage than ischemic stroke. The estimates for diabetes (7.7%) were also between the GBD (20.7%) and INTERSTROKE studies (3.9%). Some previous epidemiological studies (8, 23, 24) have produced complex results on the relationship between diabetes and intracerebral hemorrhage. However, a large community-based cohort study (25) conducted in China showed that high fasting blood glucose concentrations were associated with a higher risk of intracerebral hemorrhage. Evidence also showed that poststroke hyperglycemia was associated with larger hematoma volume, severe neurological damage and poor clinical outcome, but hemoglobin A1c was not correlated with hematoma volume or clinical outcome in patients with spontaneous intracerebral hemorrhage. INTERSTROKE showed that the relationship between diabetes and intracerebral hemorrhage in China seemed unreasonable, which was corrected in our analysis. Further investigation is needed to determine whether there is a difference between the selection of diagnostic criteria for diabetes and the association of stroke. The PAR of diabetes was larger for people living in rural areas vs. urban areas, indicating that the focus of diabetes control should be in rural areas.

For diet, different studies have used different measurement methods (7–9, 15). A high-salt diet and regular meat intake played an important role in increasing the risk of stroke. The effect of high salt intake on stroke (ischemic stroke and intracerebral hemorrhage) might be mediated *via* hypertension occurrence or control (15). Moreover, a recent study reported that the attributable risk of ischemic stroke due to dietary salt intake was probably independent of the effect of hypertension (26). It is also biologically reasonable that the meat-based diet could increase the risk of stroke (ischemic stroke and intracerebral hemorrhage), and it showed a stronger association with ischemic stroke than intracerebral hemorrhage (27, 28). The former occurs by causing atherosclerotic plaques, and interrupting blood flow to the brain, whereas the latter occurs as a result of a rupture of blood vessels (28). The meat-based diet in urban areas contributed more to the attribution risk of stroke than that in rural areas, whereas the PAR of high salt intake was greater in rural areas than in urban areas. With the rapid development of China's economy, the material standard of living in urban areas may be better. In rural areas, there was a lack of awareness and propaganda to limit salt intake in terms of dietary habits, and those who usually engaged in agricultural work preferred salt intake. The estimation of smoking should be interpreted with caution because the exposed population of smokers comprised current smokers and former smokers in our design. However, some studies have shown that compared with current smokers, former smokers had a lower risk of stroke (29, 30); even relative to never smokers, the risk of cardiovascular disease remained significantly elevated for former smokers (29), which made our analysis underestimate the PAR of smoking to stroke. Evidence indicated that mean alcohol intake had a continuously positive log-linear association with stroke risk, which was stronger for intracerebral hemorrhage (31). There was a sex difference in the association between alcohol intake and stroke because men engage in more exposure activities (31). The protective effect of moderate drinking on stroke was largely noncausal (31).

Cardiac disease was a risk factor for ischemic stroke rather than intracerebral hemorrhage, which was consistent with the INTERSTROKE results (8). However, it had a low contribution to stroke, which may be related to the low prevalence of cardiac disease. The association between high BMI and stroke still seemed unreasonable, despite conducting a number of sensitivity analyses to assess the effects of using different definitions of BMI and stroke as well as controlling for other factors that may affect BMI. As INTERSTROKE and SIREN have shown, the waist-to-hip ratio may be more representative of the relationship between obesity and stroke than BMI (8, 15). Unfortunately, we lack data related to the waist-to-hip ratio. We reported a low level of association between dyslipidemia and stroke compared with INTERSTROKE (20.5 vs. 26.8%), which may be related to our use of TC, LDL-C, HDL-C and TG instead of apolipoprotein. However, both studies demonstrated that dyslipidemia was not a risk factor for intracerebral hemorrhage.

We reported a new risk factor for high homocysteine levels. Our research showed that high homocysteine was associated with an increased risk of ischemic stroke, which has been observed in many studies (32–36). A prospective, nested case-control study indicated that a higher risk was found among patients with H-type hypertension with both high homocysteine and hypertension compared with the risk of stroke with high homocysteine and hypertension alone (37). In particular, patients with H-type hypertension may need to receive more attention and take more active measures to seek homocysteine-lowering therapy along with antihypertension therapy in Chinese populations.

A strength of our study is the representative case-control study with a large sample size (*N* = 11,729) in China's middle-income area, which gives us greater power to detect differences with fewer false negatives than INTERSTROKE (*N* = 3,987). (8). In addition, the controls for this study were all derived from the community with a catchment population of the cases, meeting the ideal design of a case-control study. Although the single-risk-factor approach highlights the potential of individual risk factors, the estimated combined PAR invalidated the assumption of independence of risk factors. Therefore, an important strength of this study is that we reported the adjusted combined PAR to explain the nonindependence of the risk factors. As we observed, the proportion of disease that can be attributed to each of the causal mechanisms of stroke can add up to more than 100%. In this analysis, we found a lower adjusted combined PAR, than the unadjusted combined PAR, which is expected and seems more plausible and stable for all individual risk factors. Adjusted PAR and unadjusted PAR do not provide conflicting information but provide a different perspective on the contribution of common risk factors. Unadjusted PAR provides a better estimate of the potential effect of complete removal of an individual risk factor on disease burden than adjusted PAR, whereas adjusted PAR might better reflect anticipated effects when multiple risk factors are modified simultaneously.

Our study has a few limitations. First, stroke cases admitted to the hospital for treatment may change the exposure level of some risk factors independently, such as physical inactivity, which may cause the exposure of the cases to be misclassified. Further research is needed on the changes in behavior patterns before and after admission to the hospital. Second, there may be selection bias in the selection of cases. Extremely severe stroke cases may be excluded because cases with mild illness or deaths from acute stroke are not easily recorded in the hospital for treatment. Third, some important risk factors for stroke in middle-income and low-income areas, such as air pollution (7) and psychological factors (8, 9, 15), were not estimated. Unfortunately, we did not measure these risk factors in the initial design.

In conclusion, the 11 common and modifiable risk factors associated with stroke that were identified will provide an important reference for evidence-based planning for stroke prevention in China and middle-income areas of other countries. There is an urgent need to improve awareness, management and control of behavioral and metabolic risk factors, particularly to promote physical activity and reduce blood pressure. In addition, the relative importance of some risk factors for specific populations is different, which provides an important basis for different populations to try to reduce the burden of stroke with targeted interventions.

## Data availability statement

The raw data supporting the conclusions of this article will be made available by the authors, without undue reservation.

## Ethics statement

The studies involving human participants were reviewed and approved by Xuanwu Hospital Capital Medical University (NO.024 [2015]). The patients/participants provided their written informed consent to participate in this study.

## Author contributions

YW: conceptualization (lead), writing-original draft (lead), formal analysis (lead), and writing-review and editing (equal). XiaoyC: data curation (equal) and software (equal). SH: conceptualization (supporting), formal analysis (supporting), and writing-review and editing (equal). HZ: methodology (lead), formal analysis (supporting), and writing-review and editing (equal). YC: conceptualization (supporting) and project administration (equal). JL: investigation (supporting). YX: data curation (equal) and project administration (equal). XiaonC: investigation (equal) and project administration (equal). LZ: resource (equal) and conceptualization (supporting). WY: conceptualization (supporting) and supervision (equal). All authors contributed to the article and approved the submitted version.

## Funding

This work was supported by Natural Science Foundation of Jiangxi Province (20202BABL216044) and National Natural Science Foundation of China (Grant No.: 81960618).

## Conflict of interest

The authors declare that the research was conducted in the absence of any commercial or financial relationships that could be construed as a potential conflict of interest.

## Publisher's note

All claims expressed in this article are solely those of the authors and do not necessarily represent those of their affiliated organizations, or those of the publisher, the editors and the reviewers. Any product that may be evaluated in this article, or claim that may be made by its manufacturer, is not guaranteed or endorsed by the publisher.
