# Supplementary material for: The impact of potentially modifiable risk factors for stroke in a middle-income area of China: A case-control study
Source: Front Public Health. 2022 Aug 19;10:815579. doi: 10.3389/fpubh.2022.815579 (PMC9437343; doi:10.3389/fpubh.2022.815579)
Supplement: Supplementary file 1 [file Table_1.DOCX]

**Supplemental Table 1.** The questionnaires and answer options for some variables

| Variable name | Answer |
| --- | --- |
| Have you ever been diagnosed with cardiac disease by doctors in secondary or higher hospitals? | 1= No 2= Yes (including atrial fibrillation, cardiomyopathy, heart failure, ischemic heart disease, rheumatic heart disease, valvular disease, and others, and abnormal ECG) |
| Smoking status (more than six months in a lifetime) | 1= Never 2= Current smoking 3= Former smoking |
| Alcohol intake | 1= Never 2= Low or moderate intake 3= High intake (more than three times a week and 100 ml each time) |
| Physical activity habits | 1= Frequently (three times or more and 0.5 h or more per week or those engaged in moderate or heavy physical labor) 2= Hardly |
| Taste preference in daily diet | 1= Salty taste 2= Light taste 3= Moderate taste |
| Meat and vegetable preference in daily diet | 1= Meat-based diet 2= Vegetable-based diet 3= Balanced meat and vegetarian diet |
