# Supplementary material for: The impact of potentially modifiable risk factors for stroke in a middle-income area of China: A case-control study
Source: Front Public Health. 2022 Aug 19;10:815579. doi: 10.3389/fpubh.2022.815579 (PMC9437343; doi:10.3389/fpubh.2022.815579)
Supplement: Supplementary file 2 [file Table_2.DOCX]

**Supplemental Table 2.** Demographic and clinical characteristics of cases

|  | N=11729 |
| --- | --- |
| Men | 6801 (58.0%) |
| Age ≤ 45 | 500 (4.3%) |
| Age | 66.76 (11.91) |
| City | 5568   (47.5%) |
| Ischemic stroke | 9880 (84.2%) |
| CT or MRI of brain | 11729 (100%) |
| Intracerebral hemorrhage | 1849 (15.8%) |
| Electrocardiogram | 6521 (55.6%) |
| Cerebral angiography | 1994 (17.0%) |
| Carotid ultrasound | 5290 (45.1%) |
