# Supplementary material for: The impact of potentially modifiable risk factors for stroke in a middle-income area of China: A case-control study
Source: Front Public Health. 2022 Aug 19;10:815579. doi: 10.3389/fpubh.2022.815579 (PMC9437343; doi:10.3389/fpubh.2022.815579)
Supplement: Supplementary file 3 [file Table_3.DOCX]

**Supplemental Table 3.** Risk factors for ischemic and intracerebral hemorrhage in men and women

|  |  | Men | | | | Women | | | |  |
| --- | --- | --- | --- | --- | --- | --- | --- | --- | --- | --- |
|  |  | Ischemic stroke | | Intracerebral hemorrhage | | Ischemic stroke | | Intracerebral hemorrhage | |  |
|  |  | OR (95% CI) | PAR (95% CI) | OR (95% CI) | PAR (95% CI) | OR (95% CI) | PAR (95% CI) | OR (95% CI) | PAR (95% CI) |  |
| Cardiac causes | | 2.99 (2.54 to 3.52) | 6.7% (5.3 to 8.3) | 0.94 (0.67 to 1.32) | -0.2% (-1.2 to 1.1) | 2.96 (2.54 to 3.44) | 7.1% (5.7 to 8.7) | 0.59 (0.41 to 0.87) | -1.6% (-2.4 to -0.5) |  |
|  |  |  |  |  |  |  |  |  |  |  |
| Hypertension | | 4.79 (4.36 to 5.27) | 60.2% (57.2 to 63.0) | 7.43 (6.13 to 9.01) | 71.9% (67.1 to 76.1) | 4.85 (4.38 to 5.36) | 60.1% (56.9 to 63.0) | 6.08 (4.97 to 7.43) | 66.5% (60.8 to 71.5) |  |
|  |  |  |  |  |  |  |  |  |  |  |
| Diabetes | | 1.88 (1.66 to 2.12) | 7.4% (5.7 to 9.2) | 2.65 (2.20 to 3.20) | 13.1% (9.9 to 16.7) | 1.79 (1.58 to 2.03) | 6.7% (5.0 to 8.5) | 3.43 (2.83 to 4.16) | 18.1% (14.3 to 22.3) |  |
|  |  |  |  |  |  |  |  |  |  |  |
| Smoking | | 0.98 (0.89 to 1.07) | -0.9% (-4.5 to 2.8) | 0.63 (0.53 to 0.74) | -17.6% (-23.3 to -11.7) | 1.32 (1.00 to 1.74) | 0.5% (0.1 to 1.2) | 0.71 (0.40 to 1.26) | -0.5% (-1.0 to 0.4) |  |
|  |  |  |  |  |  |  |  |  |  |  |
| Alcohol intake | | ... | 11.9% (5.7 to 18.4) | ... | 22.3% (10.1 to 35.0) | ... | -2.4% (-5.0 to 1.6) | ... | -1.9% (-5.9 to 6.8) |  |
|  |  |  |  |  |  |  |  |  |  |  |
| Low or moderate | | 1.50 (1.27 to 1.77) | ... | 1.92 (1.41 to 2.60) | ... | 0.69 (0.41 to 1.16) | ... | 0.73 (0.30 to 1.76) | ... |  |
|  |  |  |  |  |  |  |  |  |  |  |
| High | | 1.09 (0.91 to 1.29) | ... | 1.50 (1.09 to 2.07) | ... | 1.01 (0.60 to 1.73) | ... | 1.52 (0.62 to 3.73) | ... |  |
|  |  |  |  |  |  |  |  |  |  |  |
| Physical inactivity | | 14.25 (13.03 to 15.59) | 71.2% (69.2 to 73.2) | 13.86 (11.74 to 16.37) | 70.6% (66.8 to 74.2) | 12.47 (11.32 to 13.72) | 70.2% (67.9 to 72.3) | 12.04 (10.00 to 14.50) | 69.4% (64.8 to 73.5) |  |
|  |  |  |  |  |  |  |  |  |  |  |
| High salt intake | | 2.42 (2.20 to 2.67) | 26.2% (23.0 to 29.4) | 2.33 (1.97 to 2.76) | 25.0% (19.6 to 30.5) | 2.40 (2.17 to 2.65) | 22.7% (19.7 to 25.7) | 2.23 (1.87 to 2.67) | 20.6% (15.4 to 25.9) |  |
|  |  |  |  |  |  |  |  |  |  |  |
| Meat-based diet | | 3.74 (3.31 to 4.22) | 18.7% (16.2 to 21.3) | 3.25 (2.65 to 3.98) | 15.9% (12.2 to 20.0) | 7.82 (6.76 to 9.05) | 19.7% (17.2 to 22.5) | 6.23 (4.93 to 7.87) | 15.8% (12.4 to 19.8) |  |
|  |  |  |  |  |  |  |  |  |  |  |
| Obesity | | 0.52 (0.39 to 0.70) | -1.1% (-1.4 to -0.7) | 0.36 (0.21 to 0.63) | -1.5% (-1.9 to -0.9) | 0.47 (0.35 to 0.64) | -1.6% (-1.9 to -1.1) | 0.31 (0.16 to 0.61) | -2.0% (-2.5 to -1.2) |  |
|  |  |  |  |  |  |  |  |  |  |  |
| Dyslipidemia | | 1.87 (1.71 to 2.04) | 22.9% (19.6 to 26.3) | 1.40 (1.20 to 1.63) | 12.0% (6.3 to 17.7) | 1.96 (1.79 to 2.15) | 22.2% (19.0 to 25.5) | 1.18 (0.99 to 1.41) | 5.1% (-0.3 to 10.8) |  |
|  |  |  |  |  |  |  |  |  |  |  |
| High homocysteine | | 1.29 (1.18 to 1.41) | 10.3% (6.7 to 13.9) | 1.05 (0.90 to 1.22) | 1.9% (-4.1 to 8.2) | 1.54 (1.40 to 1.70) | 11.0% (8.4 to 13.8) | 0.82 (0.68 to 1.00) | -4.3% (-8.0 to -0.1) |  |
|  |  |  |  |  |  |  |  |  |  |  |
| Combined PAR | | ... | 96.3% (94.3 to 97.7) | ... | 96.4% (92.3 to 98.4) | ... | 95.4% (93.5 to 96.9) | ... | 94.1% (89.4 to 97.1) |  |
|  |  |  |  |  |  |  |  |  |  |  |
| Adjusted combined PAR | | ... | 66.3% (61.4 to 70.7) | ... | 63.1% (53.8 to 70.8) | ... | 63.7% (59.4 to 67.9) | ... | 57.2% (48.6 to 65.3) |  |
|  |  |  |  |  |  |  |  |  |  |  |

OR=odds ratio. PAR=population attributable risk. For alcohol intake, PAR was calculated using low or moderate + high versus never.
