# Supplementary material for: The impact of potentially modifiable risk factors for stroke in a middle-income area of China: A case-control study
Source: Front Public Health. 2022 Aug 19;10:815579. doi: 10.3389/fpubh.2022.815579 (PMC9437343; doi:10.3389/fpubh.2022.815579)
Supplement: Supplementary file 4 [file Table_4.DOCX]

**Supplemental Table 4**. Risk factors for ischemic and intracerebral hemorrhage by age group

|  |  | ≤ 55 | | | | > 55 | | | |  |
| --- | --- | --- | --- | --- | --- | --- | --- | --- | --- | --- |
|  |  | Ischemic stroke | | Intracerebral hemorrhage | | Ischemic stroke | | Intracerebral hemorrhage | |  |
|  |  | OR (95% CI) | PAR (95% CI) | OR (95% CI) | PAR (95% CI) | OR (95% CI) | PAR (95% CI) | OR (95% CI) | PAR (95% CI) |  |
| Cardiac causes | | 2.23 (1.58 to 3.12) | 1.8% (0.9 to 3.1) | 0.82 (0.45 to 1.50) | -0.3% (-0.8 to -0.7) | 2.69 (2.40 to 3.02) | 7.9% (6.6 to 9.4) | 0.72 (0.55 to 0.94) | -1.5% (-2.4 to -0.3) |  |
|  |  |  |  |  |  |  |  |  |  |  |
| Hypertension | | 5.69 (4.95 to 6.53) | 54.5% (50.3 to 58.6) | 8.96 (7.14 to 11.23) | 67.1% (61.1 to 72.4) | 3.81 (3.52 to 4.13) | 57.2% (54.5 to 59.8) | 5.64 (4.73 to 6.72) | 68.8% (64.0 to 73.1) |  |
|  |  |  |  |  |  |  |  |  |  |  |
| Diabetes | | 2.17 (1.78 to 2.64) | 6.3% (4.3 to 8.7) | 3.77 (2.93 to 4.85) | 13.8% (10.0 to 18.2) | 1.70 (1.55 to 1.87) | 7.2% (5.7 to 8.8) | 2.78 (2.38 to 3.25) | 16.4% (13.2 to 19.9) |  |
|  |  |  |  |  |  |  |  |  |  |  |
| Smoking | | 1.42 (1.20 to 1.69) | 6.3% (3.0 to 9.8) | 0.71 (0.53 to 0.95) | -4.9% (-8.1 to -0.7) | 1.26 (1.15 to 1.38) | 4.5% (2.6 to 6.4) | 0.89 (0.74 to 1.06) | -2.1% (-4.9 to 1.1) |  |
|  |  |  |  |  |  |  |  |  |  |  |
| Alcohol intake | | ... | 6.9% (0.3 to 14.9) | ... | 13.6% (1.4 to 29.6) | ... | 2.5% (-1.1 to 6.4) | ... | 5.3% (-1.7 to 14.0) |  |
|  |  |  |  |  |  |  |  |  |  |  |
| Low or moderate | | 1.44 (1.04 to 2.00) | ... | 1.83 (1.06 to 3.14) | ... | 1.11 (0.93 to 1.33) | ... | 1.20 (0.85 to 1.69) | ... |  |
|  |  |  |  |  |  |  |  |  |  |  |
| High | | 1.27 (0.91 to 1.78) | ... | 1.99 (1.15 to 3.47) | ... | 1.19 (0.99 to 1.44) | ... | 1.60 (1.12 to 2.29) | ... |  |
|  |  |  |  |  |  |  |  |  |  |  |
| Physical inactivity | | 11.47 (9.96 to 13.21) | 67.9% (64.4 to 71.1) | 9.37 (7.57 to 11.59) | 62.8% (57.0 to 68.1) | 14.00 (13.00 to 15.08) | 71.8% (70.2 to 73.4) | 15.15 (13.01 to 17.64) | 73.5% (70.2 to 76.5) |  |
|  |  |  |  |  |  |  |  |  |  |  |
| High salt intake | | 2.36 (2.04 to 2.72) | 26.1% (21.3 to 30.9) | 1.94 (1.56 to 2.41) | 19.6% (12.8 to 26.8) | 2.47 (2.28 to 2.68) | 23.2% (20.8 to 25.6) | 2.36 (2.04 to 2.72) | 21.8% (17.6 to 26.1) |  |
|  |  |  |  |  |  |  |  |  |  |  |
| Meat-based diet | | 4.98 (4.17 to 5.95) | 19.3% (16.0 to 22.9) | 4.95 (3.83 to 6.40) | 19.2% (14.5 to 24.5) | 5.49 (4.91 to 6.13) | 18.9% (16.9 to 21.1) | 4.27 (3.53 to 5.15) | 14.5% (11.6 to 17.8) |  |
|  |  |  |  |  |  |  |  |  |  |  |
| Obesity | | 0.69 (0.48 to 0.98) | -0.9% (-1.5 to -0.1) | 0.32 (0.16 to 0.62) | -1.9% (-2.4 to -1.1) | 0.46 (0.36 to 0.60) | -1.4% (-1.7 to -1.1) | 0.35 (0.21 to 0.60) | -1.7% (-2.1 to -1.1) |  |
|  |  |  |  |  |  |  |  |  |  |  |
| Dyslipidemia | | 2.20 (1.92 to 2.52) | 27.5% (22.6 to 32.4) | 1.48 (1.21 to 1.82) | 13.3% (6.3 to 20.6) | 1.98 (1.84 to 2.13) | 23.5% (20.9 to 26.2) | 1.28 (1.11 to 1.47) | 8.1% (3.4 to 12.8) |  |
|  |  |  |  |  |  |  |  |  |  |  |
| High homocysteine | | 1.93 (1.67 to 2.24) | 16.2% (12.2 to 20.4) | 1.42 (1.12 to 1.80) | 8.0% (2.4 to 14.1) | 1.28 (1.19 to 1.38) | 8.7% (6.1 to 11.3) | 0.95 (0.83 to 1.09) | -1.6% (-6.1 to 3.1) |  |
|  |  |  |  |  |  |  |  |  |  |  |
| Combined PAR | | ... | 95.7% (92.6 to 97.7) | ... | 94.9% (88.7 to 98.1) | ... | 95.8% (94.2 to 97.0) | ... | 95.7% (92.2 to 97.8) |  |
|  |  |  |  |  |  |  |  |  |  |  |
| Adjusted combined PAR | | ... | 66.5% (60.6 to 71.8) | ... | 63.9% (54.4 to 72.2) | ... | 64.3% (60.4 to 67.9) | ... | 60.6% (53.0 to 67.3) |  |
|  |  |  |  |  |  |  |  |  |  |  |

OR=odds ratio. PAR=population attributable risk. For alcohol intake, PAR was calculated using low or moderate + high versus never.
