# Supplementary material for: The impact of potentially modifiable risk factors for stroke in a middle-income area of China: A case-control study
Source: Front Public Health. 2022 Aug 19;10:815579. doi: 10.3389/fpubh.2022.815579 (PMC9437343; doi:10.3389/fpubh.2022.815579)
Supplement: Supplementary file 5 [file Table_5.DOCX]

**Supplemental Table 5.** Risk factors for ischemic and intracerebral hemorrhage in urban and rural areas

|  |  | City | | | | County | | | |  |
| --- | --- | --- | --- | --- | --- | --- | --- | --- | --- | --- |
|  |  | Ischemic stroke | | Intracerebral hemorrhage | | Ischemic stroke | | Intracerebral hemorrhage | |  |
|  |  | OR (95% CI) | PAR (95% CI) | OR (95% CI) | PAR (95% CI) | OR (95% CI) | PAR (95% CI) | OR (95% CI) | PAR (95% CI) |  |
| Cardiac causes | | 2.52 (2.18 to 2.92) | 7.1% (5.5 to 8.7) | 0.57 (0.39 to 0.84) | -2.2% (-3.2 to -0.8) | 3.51 (2.95 to 4.17) | 6.1% (4.8 to 7.6) | 1.02 (0.73 to 1.42) | 0.1% (-0.7 to -1.1) |  |
|  |  |  |  |  |  |  |  |  |  |  |
| Hypertension | | 4.31 (3.90 to 4.78) | 58.2% (54.9 to 61.3) | 5.88 (4.67 to 7.41) | 67.2% (60.6 to 72.9) | 5.20 (4.74 to 5.71) | 60.8% (58.0 to 63.5) | 7.55 (6.36 to 8.97) | 70.7% (66.4 to 74.6) |  |
|  |  |  |  |  |  |  |  |  |  |  |
| Diabetes | | 1.45 (1.28 to 1.63) | 4.7% (3.0 to 6.6) | 2.44 (1.98 to 3.01) | 13.8% (9.8 to 18.3) | 2.40 (2.12 to 2.71) | 9.0% (7.4 to 10.8) | 3.66 (3.07 to 4.35) | 15.9% (12.8 to 19.2) |  |
|  |  |  |  |  |  |  |  |  |  |  |
| Smoking | | 1.55 (1.37 to 1.75) | 6.2% (4.2 to 8.3) | 1.06 (0.82 to 1.37) | 0.7% (-2.2 to 4.2) | 1.14 (1.02 to 1.27) | 2.9% (0.5 to 5.5) | 0.72 (0.59 to 0.87) | -6.6% (-9.8 to -2.9) |  |
|  |  |  |  |  |  |  |  |  |  |  |
| Alcohol intake | | ... | 0.1% (-3.2 to 4.0) | ... | 3.6% (-3.3 to 13.5) | ... | 7.1% (1.8 to 12.9) | ... | 12.2% (2.4 to 23.7) |  |
|  |  |  |  |  |  |  |  |  |  |  |
| Low or moderate | | 0.98 (0.76 to 1.26) | ... | 1.18 (0.72 to 1.93) | ... | 1.35 (1.11 to 1.65) | ... | 1.55 (1.09 to 2.21) | ... |  |
|  |  |  |  |  |  |  |  |  |  |  |
| High | | 1.13 (0.87 to 1.47) | ... | 1.76 (1.06 to 2.93) | ... | 1.23 (0.99 to 1.52) | ... | 1.68 (1.16 to 2.43) | ... |  |
|  |  |  |  |  |  |  |  |  |  |  |
| Physical inactivity | | 17.76 (16.13 to 19.54) | 73.6% (71.5 to 75.5) | 15.60 (12.71 to 19.16) | 70.8% (66.0 to 75.1) | 9.96 (9.11 to 10.89) | 67.1% (64.9 to 69.3) | 10.84 (9.28 to 12.66) | 69.2% (65.4 to 72.7) |  |
|  |  |  |  |  |  |  |  |  |  |  |
| High salt intake | | 2.34 (2.10 to 2.61) | 16.7% (14.1 to 19.4) | 2.10 (1.71 to 2.58) | 14.2% (9.7 to 19.2) | 2.41 (2.20 to 2.64) | 29.5% (26.3 to 32.8) | 2.19 (1.88 to 2.54) | 26.1% (20.9 to 31.4) |  |
|  |  |  |  |  |  |  |  |  |  |  |
| Meat-based diet | | 6.16 (5.30 to 7.15) | 30.2% (26.5 to 34.1) | 4.31 (3.32 to 5.58) | 21.7% (16.3 to 27.8) | 4.88 (4.33 to 5.49) | 12.2% (10.7 to 13.9) | 4.50 (3.73 to 5.42) | 11.2% (8.9 to 13.7) |  |
|  |  |  |  |  |  |  |  |  |  |  |
| Obesity | | 0.38 (0.28 to 0.50) | -2.1% (-2.4 to -1.7) | 0.22 (0.10 to 0.47) | -2.6% (-3.0 to -1.8) | 0.65 (0.48 to 0.87) | -0.7% (-1.1 to -0.3) | 0.43 (0.26 to 0.72) | -1.1% (-1.5 to -0.6) |  |
|  |  |  |  |  |  |  |  |  |  |  |
| Dyslipidemia | | 1.70 (1.55 to 1.87) | 19.8% (16.3 to 23.4) | 1.41 (1.17 to 1.70) | 12.6% (5.7 to 19.7) | 2.18 (2.00 to 2.38) | 24.9% (21.9 to 27.9) | 1.33 (1.15 to 1.54) | 8.4% (4.0 to 13.1) |  |
|  |  |  |  |  |  |  |  |  |  |  |
| High homocysteine | | 1.54 (1.40 to 1.69) | 13.0% (9.9 to 16.1) | 1.10 (0.91 to 1.33) | 2.7% (-2.6 to 8.4) | 1.49 (1.36 to 1.62) | 12.8% (9.8 to 15.9) | 1.03 (0.89 to 1.20) | 0.9% (-3.5 to 5.6) |  |
|  |  |  |  |  |  |  |  |  |  |  |
| Combined PAR | | ... | 96.2% (94.3 to 97.5) | ... | 95.3% (90.1 to 98.0) | ... | 95.9% (94.0 to 97.3) | ... | 95.7% (92.0 to 97.9) |  |
|  |  |  |  |  |  |  |  |  |  |  |
| Adjusted combined PAR | | ... | 62.9% (58.5 to 67.1) | ... | 60.1% (50.7 to 68.3) | ... | 67.0% (62.8 to 70.9) | ... | 61.8% (54.3 to 68.5) |  |
|  |  |  |  |  |  |  |  |  |  |  |

OR=odds ratio. PAR=population attributable risk. For alcohol intake, PAR was calculated using low or moderate + high versus never.
